# Structural insights into a DNA polymerase reading the xeno nucleic acid HNA

**DOI:** 10.1093/nar/gkae1156

**Published:** 2024-12-03

**Authors:** Cédric Gutfreund, Karin Betz, Mikhail Abramov, Frédérick Coosemans, Phillipp Holliger, Piet Herdewijn, Andreas Marx

**Affiliations:** Department of Chemistry, University of Konstanz, Universitätsstraße 10, 78457 Konstanz, Germany; Department of Chemistry, University of Konstanz, Universitätsstraße 10, 78457 Konstanz, Germany; Department of Chemistry, University of Konstanz, Universitätsstraße 10, 78457 Konstanz, Germany; Department of Medicinal Chemistry, KU Leuven, Herestraat 49 BOX 1030, 3000 Leuven, Belgium; Department of Medicinal Chemistry, KU Leuven, Herestraat 49 BOX 1030, 3000 Leuven, Belgium; MRC Laboratory of Molecular Biology, Francis Crick Avenue, Cambridge Biomedical Campus, Cambridge CB2 0QH, UK; Department of Medicinal Chemistry, KU Leuven, Herestraat 49 BOX 1030, 3000 Leuven, Belgium; Department of Chemistry, University of Konstanz, Universitätsstraße 10, 78457 Konstanz, Germany

## Abstract

Xeno nucleic acids (XNAs) are unnatural analogues of the natural nucleic acids in which the canonical ribose or deoxyribose rings are replaced with alternative sugars, congener structures or even open-ring configurations. The expanding repertoire of XNAs holds significant promise for diverse applications in molecular biology as well as diagnostics and therapeutics. Key advantages of XNAs over natural nucleic acids include their enhanced biostability, superior target affinity and (in some cases) catalytic activity. Natural systems generally lack the mechanisms to transcribe, reverse transcribe or replicate XNAs. This limitation has been overcome through the directed evolution of nucleic acid-modifying enzymes, especially polymerases (pols) and reverse transcriptases (RTs). Despite these advances, the mechanisms by which synthetic RT enzymes read these artificial genetic polymers remain largely unexplored, primarily due to a scarcity of structural information. This study unveils first structural insights into an evolved thermostable DNA pol interacting with the XNA 1,5-anhydrohexitol nucleic acid (HNA), revealing unprecedented HNA nucleotide conformations within a ternary complex with the enzyme. These findings not only deepen our understanding of HNA to DNA reverse transcription but also set the stage for future advancements of this and similar enzymes through deliberate design.

## Graphical Abstract

**Graphical Abstract:**
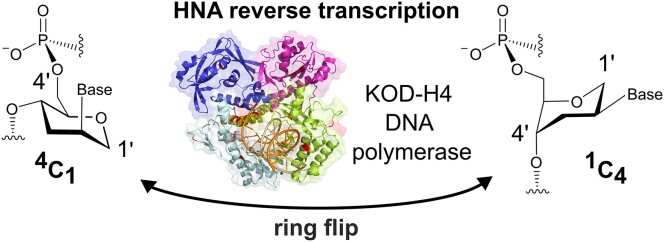

## Introduction

**Figure 1.**
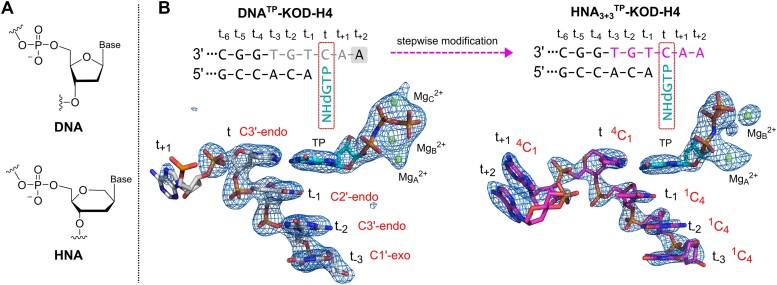
(**A**) HNA and DNA chemical structures. (**B**) Part of the sequences used to obtain DNA^TP^-KOD-H4 and HNA_3+3_^TP^-KOD-H4 are shown together with the relevant nucleotides shown as sticks. Polder maps for the displayed nucleotides as well as the TPs (including magnesium ions) are shown at 3*σ* as a blue mesh. TP stands for triphosphate. In the sequences, HNA nucleotides are highlighted in magenta. In the structures, DNA nucleotides are gray, HNA nucleotides are magenta, bound NHdGTPs are shown in cyan and Mg^2+^ ions in green. The unresolved adenosine is shaded by a gray box. Nucleotides are numbered according to the templating nucleotide t, and in the sequence, this position is indicated by a red rectangle. The assigned sugar conformations are given in red.

Xeno nucleic acids (XNAs) describe a class of synthetic polymers that are based on DNA or RNA but are altered in their sugar–phosphate backbone (1,2). A major advantage of many XNAs over DNA/RNA is their increased stability toward nucleases, making them suitable for use in many areas of molecular biology. Due to their inherent properties, such as increased chemical stability, some XNAs are also a promising basis for long-term information storage or the development of new drugs (3–7). 1,5-Anhydrohexitol nucleic acid (HNA; Figure 1A) is one of several hexose XNA analogues that have been investigated (8–12).

HNA is able to form stable right-handed antiparallel duplexes with itself, as well as with RNA and DNA, with decreasing stability following this order (13–18). These duplexes are distinguished by A-form-like helical geometries in which the 1,5-anhydrohexitol ring is in a stable chair conformation (19,20). For the use in molecular and synthetic biology as well as synthetic genetics, it is indispensable to process XNA enzymatically. To date, the number of natural DNA polymerases (pols) that are able to process XNA remains limited. One well-studied example is the DNA pol of *Geobacillus stearothermophilus*, which has been shown to possess innate XNA reverse transcriptase (RT) activity on diverse XNAs (21–23). To harness the potential of XNA, enzymes were evolved that can more effectively and specifically synthesize and reverse transcribe XNA (22,24–26). Recently, a DNA pol from *Thermococcus gorgonarius* (Tgo), termed RT-H4 (hereafter referred to as Tgo-H4), has been developed with enhanced HNA RT activity (27). Since no structural data of this enzyme (or any other) in complex with HNA are available, it remains unclear how the enzyme interacts and processes the XNA within its active site. Here, we describe the structural insight into these processes. We synthesized a series of oligonucleotides containing multiple HNA modifications near the active site and used them in co-crystallization experiments with a DNA pol with engineered HNA RT activity. We solved crystal structures of the HNA RT derived from *Thermococcus kodakarensis* (KOD) DNA pol (termed KOD-H4) in its apo state and ternary complexes with primer/template (p/t) duplexes containing six HNA nucleotides with resolutions ranging from 1.80 to 2.35 Å. The structures obtained reveal the intricacies of reverse transcription from HNA to DNA and may pave the way for the development of superior XNA-processing enzymes.

## Materials and methods

### Oligonucleotides and nucleotides

DNA oligonucleotides were purchased HPLC purified from Biomers. The oligonucleotides were used without further purification for the crystallization experiments or primer extension assays.

### HNA-containing oligonucleotides’ synthesis and characterization

HNA phosphoramidite synthesis was conducted according to the literature (28). The oligonucleotides were then assembled using the phosphoramidite approach, purified and verified by mass spectrometry. Details are depicted in the Supplementary Data.

### Protein expression and purification

The plasmid of Tgo-H4 (also termed RT-H4) was provided by the group of Philipp Holliger. The sequence of KOD-H4 was codon optimized according to Chen *et al.* (29), purchased from Azenta Life Sciences and cloned into pET21b(+) (Novagen). KOD plasmids were transformed into OverExpress C43(DE3) (BioCat) and Tgo plasmids were transformed into Rosetta™ (DE3). They were overexpressed by inoculating LB media (2 l) containing 100 mg/l carbenicillin (KOD/Tgo-H4 variants) to an OD_600_ of 0.1. Cells were grown at 37°C and 180 rpm to an OD_600_ of 0.6–0.8, and then the protein expression was induced by addition of 1 mM Isopropyl β-D-1-thiogalactopyranoside (IPTG) for KOD variant or 200 μg/l anhydrotetracycline for Tgo variant, which was followed by overnight incubation at 37°C and 180 rpm. After harvesting the cells at 4000 rpm (30 min), the pellets were resuspended in RT lysis buffer (20 mM Tris–HCl, pH 7.4, 200 mM NaCl, 2 mM MgSO_4_, 0.1% Triton^®^ X-100, 0.5% NP-40) and the protease inhibitors aprotinin (10 mg/ml), leupeptin (1 mg/ml) and pefabloc (100 mg/ml) were added. Cells were lysed by the addition of hen egg white lysozyme (0.1 mg/l) and incubation at 37°C for 1 h, followed by heat denaturation at 60°C for 30 min. The lysates were cleared by ultracentrifugation (24 000 rpm, 60 min, 10°C) and filtered through a 0.45-μm sterile filter. The supernatant was applied to a 5-ml Hi-Trap™ Q HP column (GE Healthcare) and washed with 35% RT-high buffer [RT-low buffer: 20 mM Tris–HCl, pH 7.4, 50 mM NaCl, 10% (w/v) glycerol; RT-high buffer: 20 mM Tris–HCl, pH 7.4, 1 M NaCl, 10% (w/v) glycerol]. The flowthrough and wash fractions containing the pol variant were then applied to a Hi-Trap™ Heparin HP column (GE Healthcare), which was washed again with 35% and finally eluted with 65% RT-high buffer. The elution fraction of 65% RT-high was concentrated to <5 ml at room temperature with repeated resuspension every 10 min (4000 rpm) using a 50 000 kDa Vivaspin (Sartorius). The sample was then applied to a HiLoad 16/600 Superdex 200pg (GE Healthcare) size exclusion chromatography column using RT-storage buffer [20 mM Tris–HCl, pH 7.4, 200 mM NaCl, 1 mM 2-mercaptoethanol, 0.1 mM EDTA, 10% (v/v) glycerol]. Pure fractions (as determined by sodium dodecyl sulfate–polyacrylamide gel electrophoresis) were pooled and concentrated at room temperature with repeated resuspension every 10 min (4000 rpm) using a 50 000 kDa Vivaspin (Sartorius) to final concentrations of 8–20 mg/ml as determined by either Nanodrop (Thermo Fisher) or Bradford assay. They were then aliquoted, snap frozen in liquid nitrogen and stored at −80°C.

### Primer extension assays

Primers and reference DNA products [DNA-Primer 5′-d(GAC CAC GGC CA)-3, DNA-Template 5′-d(AAC TGT GGC CGT GGT C)-3′] were 5′-radioactively labeled using [γ-^32^P]-ATP (Hartmann Analytic) and T4 polynucleotide kinase (New England Biolabs). HNA template [HNA: 5′-h(AAC TGT GGC CGT GGT C)-3′] was used for all HNA experiments. Reactions were performed in 10 μl volumes. Primers and templates were annealed by heating to 95°C for 2 min and gradually cooling to 4°C. Final concentrations that were used in all reactions are as follows: 1× ThermoPol^®^ reaction buffer [New England Biolabs; 20 mM Tris–HCl, 10 mM (NH_4_)_2_SO_4_, 10 mM KCl, 2 mM MgSO_4_, 0.1% Triton^®^ X-100, pH 8.8 @ 25°C], 15 nM ^32^P-labeled primer, 15 nM template, 100 μM dNTPs and 0.1 μM respective pol. Reactions were carried out at 55°C for 10 or 60 min and stopped by the addition of 20 μl of stopping solution [80% (v/v) formamide, 20 mM EDTA, 0.25% (w/v) bromophenol blue, 0.25% (w/v) xylene cyanol], followed by heat denaturation at 95°C for 2 min and analysis by 20% urea polyacrylamide gel electrophoresis. The gels were imaged by phosphorimaging using a Typhon FLA 7000 (GE Healthcare).

### Fluorescence polarization assay

The concentration of purified pol variants was determined by Bradford assay and diluted to 2 μM stock concentration in 1× ThermoPol^®^ reaction buffer [20 mM Tris–HCl, 10 mM (NH_4_)_2_SO_4_, 10 mM KCl, 2 mM MgSO_4_, 0.1% Triton^®^ X-100, pH 8.8 @ 25°C]. A 5′-6-fluorescein-labeled DNA primer [5′-6-FAM-d(GAC CAC GGC C)-3] was annealed with the respective templates [DNA-Template 5′-d(AAC TGT GGC CGT GGT C)-3′, HNA 5′-h(AAC TGT GGC CGT GGT C)-3′] by heating to 95°C for 2 min and gradually cooling to 25°C. Concentrations were adjusted to 10 nM primer and 100 nM template in 1× ThermoPol^®^ reaction buffer. Titration experiments were performed at 25°C on a Tecan Spark (Thermo Fisher). All measurements were performed in triplicates with three replicates each and a total volume of 45 μl by combining equal volumes of p/t (final concentration of labeled primer 5 nM) with the respective pols in varying concentrations. After mixing, the plates were incubated at 25°C for 20 min before measuring the fluorescence. Samples were excited at 485 nm (20 nm bandwidth) and detected at 535 nm (20 nm bandwidth). Fluorescence intensities were corrected for sample dilution and inner filter effect (30). Dissociation constants $K_{\rm D}^{{\rm app}}$ were calculated from weighted averages, with the weights taken from reciprocal standard deviations squared by dose–response analysis (four parameters) using GraphPad Prism version 9.4.1 for Windows (GraphPad Software, San Diego, CA, USA, www.graphpad.com).

### Crystallization and structure determination

#### Crystallization conditions

Primers and templates [13mer-DNA-Primer 5′-d(GAC CAC GGC CAC A)-3′, DNA-Template 5′-d(AAC TGT GGC CGT GGT C)-3′, HNA_3+3_-Template 5′-d(A*A*C* T*G*T* GGC CGT GGT C)-3′, asterisk indicating HNA nucleotides] were dissolved in high-purity (Milli-Q) water and stored at −20°C as 6 mM solutions. Primers and templates were then mixed in a ratio of 1:1 and were heated to 95°C for 5 min and then gradually cooled to 10°C. Afterward, RT-low buffer [20 mM Tris–HCl, pH 7.4, 50 mM NaCl, 10% (w/v) glycerol] and the respective pol were added to final concentrations of either 3.5 or 5.0 mg/ml; 20 mM MgCl_2_ or 10 mM MgCl_2_ and MnCl_2_ each was added. The solution was incubated at 55°C for 30 min. For ternary crystals, a 10-fold molar excess of 2′-deoxyguanosine-5′-[(α,β)-imido]triphosphate (Jena Bioscience) was added, and the solution was incubated for an additional 30 min at 30°C. Finally, all solutions were incubated at 4°C for 45 min and filtered through a 0.1-μm sterile filter (Ultrafree Centrifugal Filters, Millipore). Crystallization set-ups were performed using the sitting drop vapor diffusion method with a Gryphon robot (Art Robbins Instruments) and commercially available screens. The protein was mixed with the reservoir solution in ratios of 1:2, 1:1 and 2:1, resulting in a final drop size of 0.8–0.9 μl. Crystals were either directly harvested and flash frozen or cryo-protected using 20% (v/v) glycerol or ethylene glycol mixed with the respective crystallization conditions and subsequently stored in liquid nitrogen until shipping.

#### Data collection

The synchrotron data were collected at beamlines P13 and P14 operated by EMBL Hamburg at the PETRA III storage ring (DESY, Hamburg, Germany) (31). Raw data files have been uploaded to SBGrid.

#### Structure determination

Data were processed using the XDS package and the XDSGUI graphical interface (https://strucbio.biologie.uni-konstanz.de/xdswiki/index.php/XDSGUI) (32). The structures were solved by molecular replacement against published KOD DNA pol structures (PDB: 5OMF or 4K8Z). Structure refinement was performed using phenix (33). Model building was done in Coot (34,35). Model quality was evaluated by the MolProbity (http://molprobity.biochem.duke.edu) and the PDB validation server (https://validate-rcsb-1.wwpdb.org) (34–36). Molecular figures were created with PyMOL as well as all-atom root-mean-square deviation (RMSD) value calculations (37). DNA conformations were analyzed using the Web 3DNA server (http://web.x3dna.org/) (38). For the refinement of HNA nucleotides, the restraints (cif) files were generated using phenix.elbow (39) and edited manually using phenix.reel. To not restrain dihedral angles and angles within the hexose ring in refinement, the variance values (value_angle_esd) for the respective dihedral angles and angles were set to 180° and 10°, respectively. All structures were deposited at the PDB database (PDB accession codes: 8S87, 9EMI and 8S84).

## Results

### Transferring HNA RT activity to KOD DNA pol

The aim of this study was to structurally characterize the mechanism of the laboratory-evolved reverse transcriptase Tgo-H4 (previously RT-H4) in interaction with its substrates, an HNA/DNA primer/template duplex and a bound triphosphate (27). As previous attempts to crystallize Tgo-H4 have been unsuccessful, we transferred its 18 mutations into the backbone of KOD DNA pol, which shares 93% sequence identity with the Tgo DNA pol (see Supplementary Figure S1 for sequence alignment) and had been successfully crystallized by us before (40–43).

The generated KOD-H4 enzyme includes the following mutations: V93Q, I114T, D141A, E143A, S383K, K429G (E429G in Tgo-H4), F445L, A485L, Y493V (F493V in Tgo-H4), Y496H, Y497M, Y499F, A500E, R501N (K501N in Tgo-H4), I521L, E664K, K726R and N735K. KOD-H4 was successfully expressed and purified in *Escherichia coli*. Both Tgo-H4 and KOD-H4 are exonuclease-deficient (D141A, E143A). Just as Tgo-H4, Kod-H4 exhibits HNA reverse transcription activity, as was tested in ^32^P-based primer extension assays (Supplementary Figure S2A and B). Furthermore, the binding affinity of both mutants was determined via fluorescence polarization for DNA/DNA and DNA/HNA duplexes (Supplementary Figure S2C). Since both mutants exhibit Hill slopes in the range of 1.6–2.0 (Supplementary Table S1), which highlights potential cooperative binding events, binding affinities were determined as apparent *K*_D_ values ($K_{\rm D}^{\rm app}$). Both Tgo-H4 and KOD-H4 exhibit similar binding affinities in the nanomolar range for DNA as well as HNA (Supplementary Table S1). Tgo-H4 shows slightly higher affinities to both DNA (Tgo-H4: 10.01 nM; KOD-H4: 12.50 nM) and HNA (Tgo-H4: 61.47 nM; KOD-H4: 80.19 nM). In addition, in both enzymes, the binding affinity to HNA is approximately six times lower compared to DNA. These results show that HNA RT activity can be directly transferred from Tgo-H4 by mutating KOD DNA pol.

### Crystal structures of KOD-H4 in complex with HNA

Next, we set out to crystallize KOD-H4 with modified templates in order to gain structural insights into the processing of HNA and obtained several crystal structures. HNA phosphoramidites were synthesized according to the literature (28) and used to synthesize several HNA-containing templates. At first, we crystallized KOD-H4 in a ternary DNA complex (termed DNA^TP^-KOD-H4, PDB: 8S84; Figure 1B, left) with a natural DNA/DNA duplex [13mer-DNA-Primer 5′-d(GAC CAC GGC CAC A)-3′, DNA-Template 5′-d(AAC TGT GGC CGT GGT C)-3′] and a bound non-hydrolyzable dGTP analogue (NHdGTP). This structure was used to assess the overall structural changes as well as any mutation-specific changes in its conformation compared to the ternary DNA wild-type KOD structure [DNA^TP^-KOD-wt, PDB: 5OMF (41)]. Additionally, we successfully crystallized the enzyme in its apo form (termed apo-KOD-H4, PDB: 8S87). Crystal conditions are given in Supplementary Table S2 and crystallographic data and refinement statistics are summarized in Supplementary Table S3. As attempts to crystallize the pol in complex with a fully modified DNA/HNA duplex have so far been unsuccessful, we decided to increase the modification density gradually by stepwise replacement of template residues with HNA nucleotides. Replacement was started at the last duplexed position in the ternary complex at the post-insertion site (position t_−1_, where t stands for the templating position) and expanded toward the duplex and the 5′ single-stranded (ss) template region (Figure 1). We crystallized KOD-H4 successfully with a range of templates containing multiple HNA nucleotides with up to six HNA nucleotides [HNA_3+3_-Template 5′-d(A*A*C* T*G*T* GGC CGT GGT C)-3′, asterisk indicating HNA nucleotides]. In this process, we obtained a ternary complex structure in an active state (termed HNA_3+3_^TP^-KOD-H4, PDB: 9EMI; Figure 1B, right, and Supplementary Table S3) with HNA modifications in positions t_+2_ to t_−3_ (three nucleotides in the duplexed region, one in the templating position and two in the ss overhang) that was similar to a natural complex.

In instances where the template contained four, five or six HNA nucleotides within the duplexed region, we obtained structures in which the p/t duplex was no longer bound near the active site of the enzyme, but in an unproductive manner.

**Figure 2.**
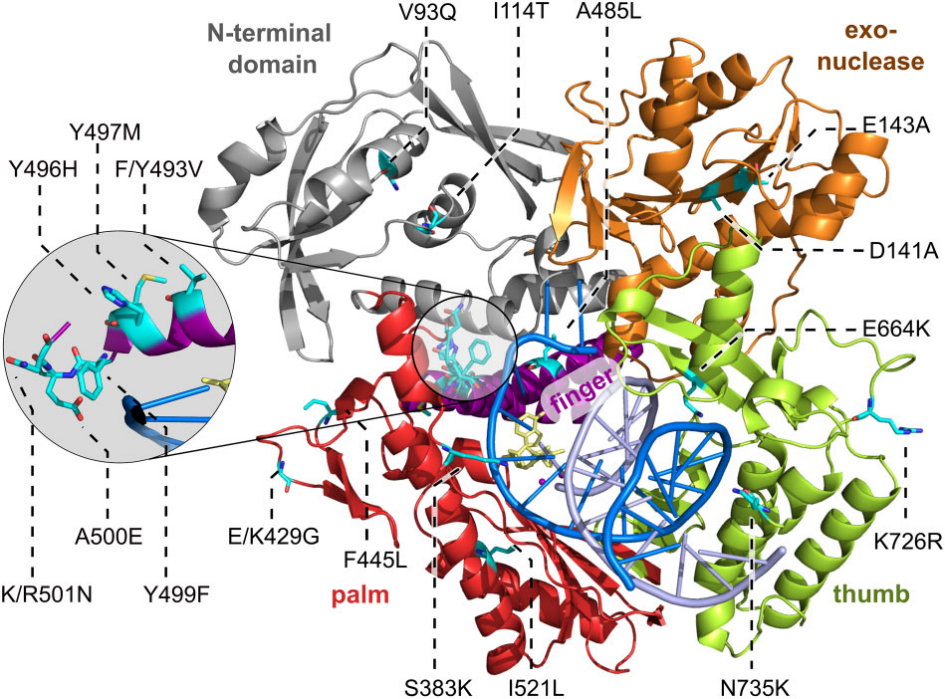
Crystal structure of HNA_3+3_^TP^-KOD-H4 ternary complex with HNA_3+3_ template (blue), DNA primer (light blue) and an NHdGTP (yellow). Domains are labeled bold in colors as follows: N-terminal (gray), exonuclease (orange), thumb (green), palm (red) and finger (purple). Mutations are shown as cyan sticks. A close-up of the finger domain shows the location of the HNA-specific mutations (Y496H, Y497M, Y499F, A500E, R501N) established by Houlihan *et al.* (27).

Next, we will describe the structures in detail and discuss features of the mutant enzyme as well as DNA/HNA binding and conformation. All structures of KOD-H4 show the typical features of an archaeal DNA pol resembling a right hand with five distinct subdomains: N-terminal (1–130, 327–368), 3′–5′-exonuclease (131–326), palm (369–449, 500–587), finger (450–499) and thumb (588–774). The structural features of the enzyme are highlighted in Figure 2, which shows the ternary HNA_3+3_^TP^-KOD-H4 complex with the template containing six HNA nucleotides and an incoming TP (6). All mutations are shown as cyan sticks and are labeled. The overall structure is not affected by the 18 introduced mutations; however, noticeable local differences are evident when comparing the ternary complexes to the respective DNA^TP^-KOD-wt structure as described further below.

### Ternary complexes

**Figure 3.**
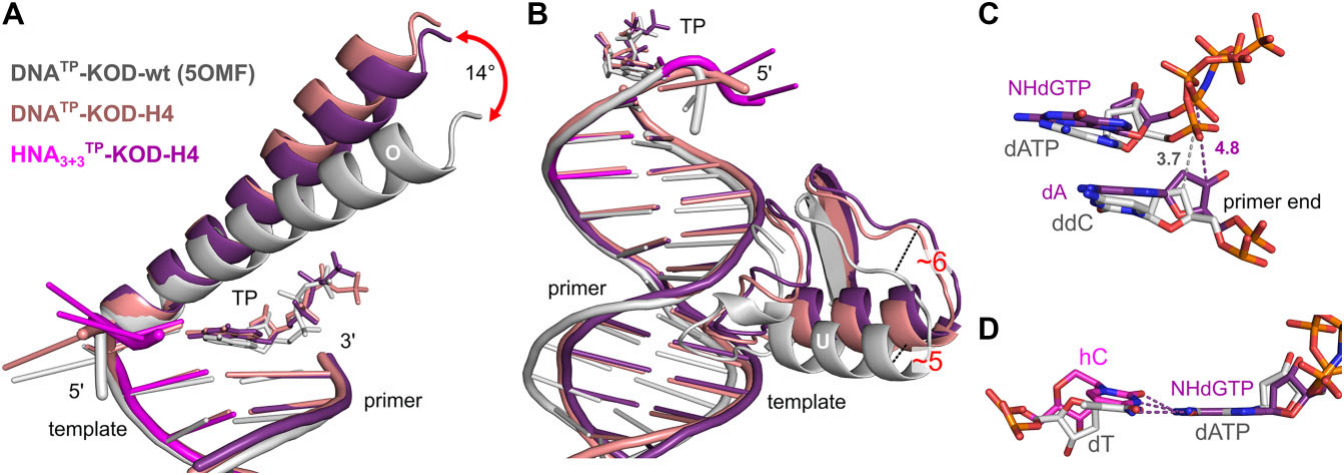
Overlay of DNA^TP^-KOD-wt (gray) (41), DNA^TP^-KOD-H4 (salmon) and HNA_3+3_^TP^-KOD-H4 (purple). HNA nucleotides are shown in magenta. Distances (Å) and angles were measured from corresponding C-α atoms. (**A**) Positioning of the finger domain O-helix relative to the p/t duplex with the incoming TPs. (**B**) Thumb domain displacement is highlighted for the U-helix. (**C**) Positioning of the incoming TP relative to the primer end in DNA^TP^-KOD-wt and HNA_3+3_^TP^-KOD-H4. The distance of the primer end to the α-phosphate is indicated by dashed lines. (**D**) Overlay of nascent base pairs of DNA^TP^-KOD-wt and HNA_3+3_^TP^-KOD-H4. Superimposition was done based on the TP nucleobase. Base pairing hydrogen bonds for HNA_3+3_^TP^-KOD-H4 are indicated by dashed lines.

To gain insights into substrate binding and catalysis, we analyzed the two ternary complexes DNA^TP^-KOD-H4 and HNA_3+3_^TP^-KOD-H4, both complexed with NHdGTP. In the following, the ternary complexes are first compared with the ternary DNA^TP^-KOD-wt structure (PDB: 5OMF) with a bound DNA/DNA duplex and dATP and afterward with each other. Major differences between the DNA^TP^-KOD-wt structure and KOD-H4 are present in the thumb and the finger domain (Figure 3). While the thumb domain closes upon binding of a p/t duplex, the finger domain (consisting of the O- and the N-helix) closes tightly by a rotation movement upon binding of a correct triphosphate in the DNA^TP^-KOD-wt structure (41).

Most striking is that in both ternary KOD-H4 structures (HNA_3+3_^TP^-KOD-H4 and DNA^TP^-KOD-H4) the finger domain closes only partially upon TP binding. While the finger domain undergoes a closure of ∼25° from binary to ternary complex in the wt structure, it closes only by rotation around 7° and 11° in the HNA_3+3_^TP^-KOD-H4 and DNA^TP^-KOD-H4 structures, respectively (Figure 3A). Apart from changes in the finger domain, the complete thumb domain is shifted by ∼5–6 Å when comparing to the DNA^TP^-KOD-wt structure (Figure 3B). This goes along with a slightly shifted p/t duplex in that region (see also Supplementary Figure S4D and F). Further downstream (nucleotides t_−11_ to t_−16_ and p_−10_ to p_−13_), the p/t duplex is not bound within the enzyme anymore, making it more flexible in the three structures as visualized in the superimposition in Supplementary Figure S4 (for interaction maps, see Supplementary Figure S5).

As in the wt, the bound TP is located in the TP binding site in both ternary KOD-H4 structures. Although located in the same pocket, the TPs in both ternary KOD-H4 structures are shifted toward the O-helix. At the same time, the 3′-ends of the primers are also shifted (see Supplementary Figure S4D and F). These shifts correlate with the open finger domain. The TPs are base paired to the templating nucleotides but adopt some different base pairing geometries (Supplementary Figure S4D and F). Especially, the co-planarity of the template nucleotide and the incoming TP is affected. While in the wt the nucleobases of the base pair are co-planar within the incorporation site, a tilt is visible in both ternary KOD-H4 structures. This tilt is more pronounced in HNA_3+3_^TP^-KOD-H4 compared to DNA^TP^-KOD-H4 (see Supplementary Figure S6). The TPs in HNA_3+3_^TP^-KOD-H4 as well as DNA^TP^-KOD-H4 interact with several residues in their vicinity (Supplementary Figure S5) but are not as tightly bound as the TP in the closed complex DNA^TP^-KOD-wt, which is supported by the relatively high *B*-factors of the TPs. Also, the arrangement and composition of the catalytic metal ions differ (Supplementary Figure S3A–C). The distance of the primer end to the α-phosphate of the incoming TPs (measured from C3′; Supplementary Figure S4D and F) is 4.8 Å for HNA_3+3_^TP^-KOD-H4 and 5.0 Å for DNA^TP^-KOD-H4 and therefore significantly higher than the distance in the wt (3.7 Å). Comparing the two ternary crystal structures of KOD-H4 to each other highlights their strong structural similarity and shows the close similarity of the binding of HNA and DNA duplexes within the enzyme’s active site (Supplementary Figure S4A). The RMSD of HNA_3+3_^TP^-KOD-H4 and DNA^TP^-KOD-H4 is 0.809 Å. Overlay of the duplexes alone leads to an RMSD of 0.498 Å.

### HNA-specific interactions

**Figure 4.**
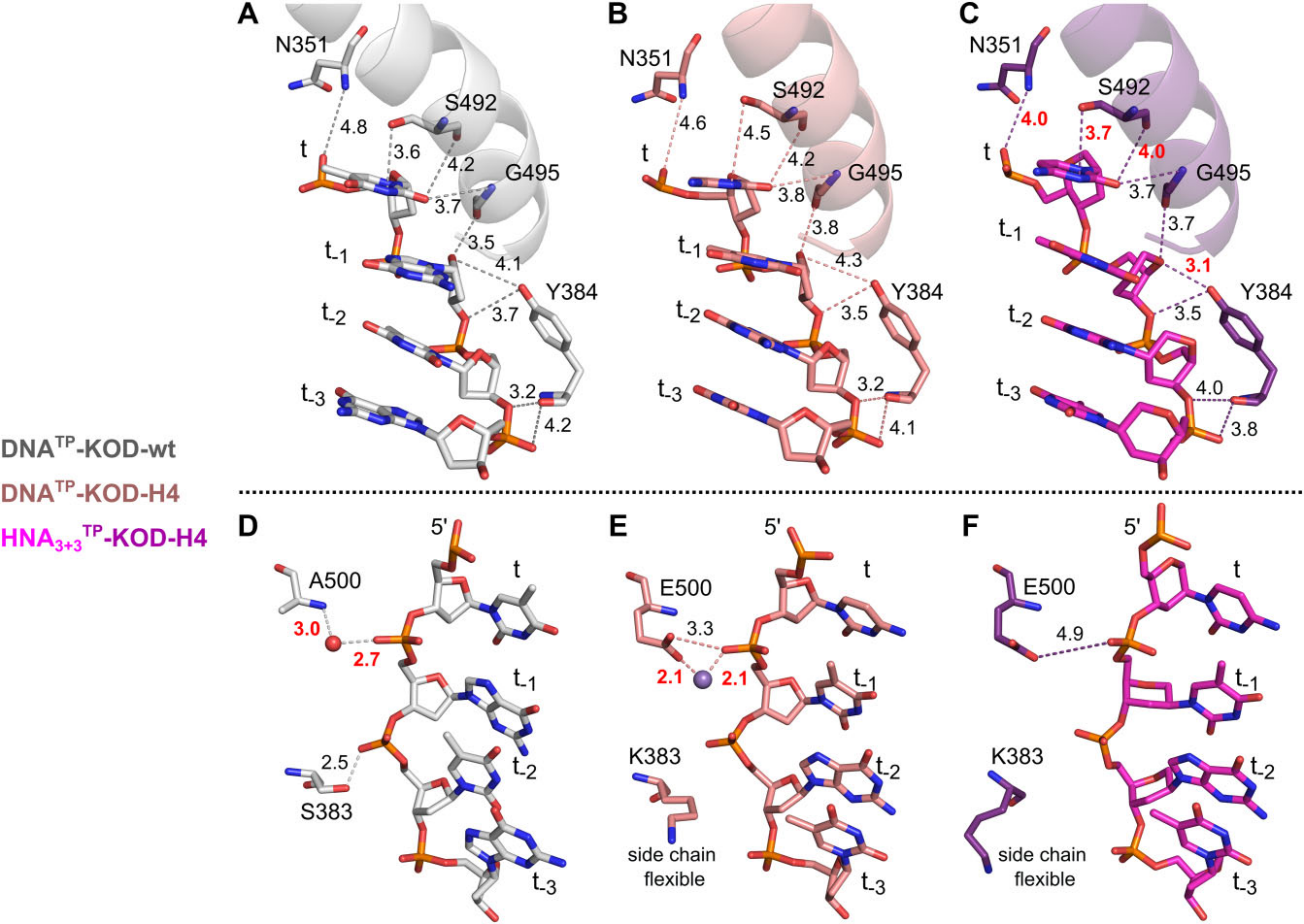
Differences in template interactions in DNA^TP^-KOD-wt (gray), DNA^TP^-KOD-H4 (salmon) and HNA_3+3_^TP^-KOD-H4 (purple) are shown. HNA nucleotides are shown in magenta. Polar interactions are shown as dashed lines and distances are given in Å. (**A**–**C**) Closer interactions in the complex HNA_3+3_^TP^-KOD-H4 compared to DNA^TP^-KOD-H4 or DNA^TP^-KOD-wt are shown in bold red letters. (**D**–**F**) Loss of interactions of the HNA template of HNA_3+3_^TP^-KOD-H4. A water molecule is shown as a red sphere and a manganese ion as a purple sphere. Note that many p/t interactions are common to all three complexes and are not shown in this figure.

To further understand how KOD-H4 is able to read HNA, we examined the polar interactions of its ternary complexes with DNA and HNA (Supplementary Figure S5) in comparison with the DNA^TP^-KOD-wt structure (41). Therefore, we focused on HNA-specific interactions to the templating nucleotide (t) and the three following nucleotides (t_−1/−2/−3_). The differences between the interaction patterns of the three ternary complexes (DNA^TP^-KOD-wt, HNA_3+3_^TP^-KOD-H4, DNA^TP^-KOD-H4) are summarized in Figure 4.

Several polar contacts to the HNA are detected in HNA_3+3_^TP^-KOD-H4 that are closer than those in the DNA^TP^-KOD-H4 or the DNA^TP^-KOD-wt structure (Figure 4A–C). The side chain and backbone of S492 (O-helix) are positioned closer to the nucleobase of the templating nucleotide compared to KOD-H4 bound to DNA (purple versus salmon in Figure 4). An HNA-specific interaction may be established between the backbone of N351 (K-helix, palm) and the phosphate of the templating nucleotide probably stabilizing the sterically more demanding nucleotide at that position. Further, Y384 (located in the flexible linker region in the palm domain between the L-helix and β-sheet 18) can interact with the HNA in positions t_−1/−2/−3_. Here, the interaction with the ring oxygen is significantly closer to the hexitol ring compared to the ribose rings (3.1 Å versus 4.1 or 4.3 Å). In contrast to these specific interactions of KOD-H4 to the HNA template, other interactions of the template are not observed in both KOD-H4 crystal structures when compared to the wt (Figure 4C). Interestingly, both missing interactions stem from two of the mutation sites of KOD-H4, S383K and A500E in the palm domain. While in the wt S383 interacts with the phosphate backbone (2.5 Å distance), the mutated lysine residues in both KOD-H4 structures are flexible with poor side chain electron density leading to a loss of interaction. Further, in KOD-wt, the backbone of A500 can interact with the phosphate backbone via a water molecule. In KOD-H4, A500 is mutated to a glutamate, which—at least in the structure of DNA^TP^-KOD-H4—seems to establish a similar interaction via its side chain either directly or through a bound manganese ion. In HNA^TP^-KOD-H4, no ion or water molecule could be found at that position and an interaction might be weak.

### HNA conformational adaptation

**Figure 5.**
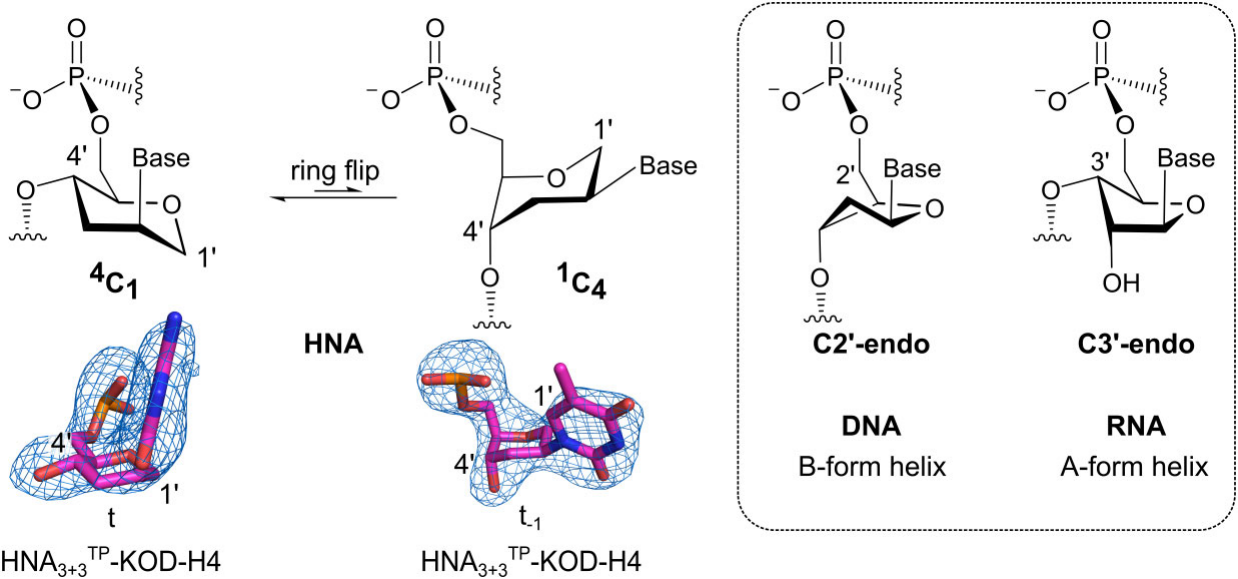
Scheme of the ring flip in hexitol nucleotides with respective examples of observed HNA conformations in HNA_3+3_^TP^-KOD-H4 in comparison to the most prevalent DNA/RNA conformations for the respective B/A-form helix geometries. Omit maps for nucleotides are shown at 3*σ* as mesh.

Structural studies of HNA/HNA and HNA/RNA structures show that the six-membered 1,5-anhydrohexitol ring of HNA usually adopts a ^4^C_1_ chair conformation with the nucleobase in an axial position (C4′ atom pointing up in the chair and C1′ pointing down; see Figure 5) (19,20,44,45). The pyranose ring mimics the ribose conformation in a C2′-*exo*/C3′-*endo* position (Figure 5); this is due to the structural rigidity of the six-membered ring (19,45).

In comparison, in DNA or RNA, the C3′-*endo* ribose conformation is found in A-form helices, whereas the C2′-*endo* conformation is mainly found in B-form helices. We assigned the HNA chair conformations in our crystal structures (Figure 1B), based on the electron densities and considering resolution. Surprisingly, we found that several HNA nucleotides do not adopt the ^4^C_1_ conformation that HNA adopts in all other published structures (46). Instead, in some cases, it adopts an unfavored ^1^C_4_ conformation (Figure 5). The ^1^C_4_ conformation is identified in the three duplexed nucleotides (t_−1_ to t_−3_) in HNA_3+3_^TP^-KOD-H4. These three nucleotides are properly base paired to their partner and located within the double helix that is tightly bound near the active site of the enzyme. In contrast, single-stranded HNA nucleotides or the one that does not show proper base pairing in the templating position or those in the unproductive complexes (data not shown) adopt the common ^4^C_1_ conformation. The flipped ^1^C_4_ conformation mimics the C3′-*exo* ribose conformation, which, together with the preferred DNA C2′-*endo* conformation, are both south sugar conformations that are mainly associated with B-form DNA helices (Figure 5) (47,48). The conformational flexibility of HNA is also evident when looking at the phosphate distances within the primer or template chains, and comparing them to the distances of natural B-form (∼7.0 Å) and A-form helices (∼5.9 Å) (20,47,49–51). In our crystal structures, the phosphate distances assume varying values of 5.8–6.8 Å (Supplementary Table S4), which are in stark contrast to the previously published HNA duplex structures where the values range from 5.5 to 5.8 Å (19,45).

## Discussion

With the aim of elucidating the reverse transcription of HNA into DNA on a molecular basis, we report here structural data of an evolved XNA RT that can read HNA and transcribe it into DNA. We crystallized the enzyme with a template containing six HNA nucleotides and analyzed the crystal structures for HNA-specific adaptations.

For both HNA_3+3_^TP^-KOD-H4 and DNA^TP^-KOD-H4, we observed open ternary complexes in which the distance from the primer end to the α-phosphate of the incoming TPs (4.8 and 5.0 Å, respectively) is significantly higher than the distance in the DNA^TP^-KOD-wt (3.7 Å). This suggests that prior to catalysis, another conformational change may be required to bring the substrates closer together. This could be achieved either by a movement of the TP (possibly initiated by a closure of the finger domain) or by a displacement of the p/t duplex or the primer alone. Similar open ternary complexes have been observed for two KOD mutants: (i) RTX (52) (RT activity, PDB: 6WYA) bound to a DNA/DNA duplex and a non-hydrolyzable analogue of dATP and (ii) KOD-RI (53) [α-l-threofuranosyl nucleic acid (TNA) pol, PDB: 5VU7] bound to a modified p/t complex (52,54). In addition, the templating nucleotide in HNA_3+3_^TP^-KOD-H4 is tilted, which might stem from the expanded bulk of the 1,5-anhydrohexitol ring compared to the ribose. Chim *et al.* observed deviations in co-planarity of the nucleotide within their TNA synthesizing KOD-RI mutant and argued that this could make the enzyme less efficient by slowing down the rate of catalysis due to the non-optimal geometry (53).

To date, only crystal structures and solution nuclear magnetic resonance structures of HNA/RNA or HNA/HNA duplexes are available, and no structures of DNA/HNA duplexes beyond a modeled structural proposal have been published (19,20,44,45). The structural studies all indicate that the six-membered 1,5-anhydrohexitol ring of HNA leads to a decrease in XNA flexibility, due to the rigid chair conformation. The chair mimics the furanose ribose ring in a C2′-*exo*/C3′-*endo* position (Figure 5), resulting in a pre-organization of HNA that favors the formation of A-type helices in duplexes with RNA or with itself (45). These duplexes are characterized by canonical hydrogen bonding as well as efficient base stacking, where the HNA moieties are in a ^4^C_1_ conformation and the nucleobase is positioned axially due to steric constraints imposed by the unshared electron pairs of the ring oxygen (45). In DNA or RNA, ideal A-form helices have a predominant C3′-*endo* ribose conformation, whereas in B-form helices C2′-*endo* is favored.

In contrast to the previously observed HNA conformations, we observe an enzyme-induced conformational change of an HNA-containing nucleic acid duplex in which the six-membered 1,5-anhydrohexitol ring undergoes a ring flip to the ^1^C_4_ conformation. This leads to the adoption of a more B-form helical geometry (also in terms of the phosphate–phosphate distances) within the active site of the pol, which may be important for the acceptance of the HNA modification. We propose that several criteria must be met to force the HNA nucleotides into the unusual conformation: (i) the HNA nucleotide must be bound near the active site of the enzyme and (ii) the nucleotide must be positioned within a nucleic acid duplex and establish proper base pairing. If these criteria are not met, the canonical ^4^C_1_ conformation is observed.

As B-family pols, such as KOD, favor B-form helical geometries in their active site, in contrast to A-family pols that favor A-form, we hypothesize that the HNA conformation in the duplex must change in order to be properly bound (41,52,55–57). This conformational change, however, is energetically unfavorable (46). Therefore, we suggest that it is more difficult for KOD-H4 to stably bind p/t duplexes containing more HNA nucleotides, which would explain why we could not obtain crystals of productive complexes with templates containing more HNA nucleotides. *In silico* modeling of the sugar puckering and potential surface energy of hexitol nucleosides by Mattelaer *et al.* points to the low flexibility of HNA, which is characterized by high transition states between the local energetic minima. Energies in the range of 3.7–5.5 kcal/mol are required to induce the ring flip in the nucleosides (46). Our findings help to demonstrate that HNA may not be as rigid as previously assumed in the context of binding to enzymes (13,44,45,58). This is the first time that the energetically unfavored ^1^C_4_ HNA conformation has been observed in a nucleic acid duplex. Interestingly, the same conformation is also adopted by a single 5-iodouracil anhydrohexitol nucleoside bound to HSV-1 thymidine kinase. Also here, it is assumed that the viral enzyme induces the conformational change (59).

In summary, our data provide a basis for the optimization and rational design of DNA pols, especially since better tools are needed to exploit the immense potential of XNAs for advanced applications. One promising approach could be to use these findings to engineer A-family pols for reverse transcription of HNA, since they generally bind duplexes of the A-form near the active site.

## Supplementary Material

gkae1156_Supplemental_File

## Data Availability

Crystallographic raw data files have been uploaded to SBGrid. All protein structures have been deposited at the PDB database (PDB accession codes: 8S87, 9EMI and 8S84).
