# Supplementary material for: Structural insights into a DNA polymerase reading the xeno nucleic acid HNA
Source: Nucleic Acids Res. 2024 Dec 3;53(1):gkae1156. doi: 10.1093/nar/gkae1156 (PMC11724289; doi:10.1093/nar/gkae1156)
Supplement: gkae1156_Supplemental_File [file gkae1156_supplemental_file.pdf]

Structural Insights into a DNA Polymerase Reading the Xeno Nucleic Acid HNA

AUTHORS

Cédric Gutfreund[1], Karin Betz[1], Mikhail Abramov[1,2], Frédérick Coosemans[2], Phillip Holliger[3], Piet Herdewijn[2], Andreas Marx*[1]

[1] Department of Chemistry, University of Konstanz; Universitätsstraße 10, 78457 Konstanz, Germany.
[2] Department of Medicinal Chemistry, KU Leuven, 3000, Leuven, Belgium
[3] MRC Laboratory of Molecular Biology, Francis Crick Avenue, Cambridge Biomedical Campus, Cambridge CB2 0QH, UK

* To whom correspondence should be addressed. Tel: +49 7531885139; Email: andreas.marx@uni-konstanz.de

SUPPLEMENTARY DATA

MATERIAL AND METHODS

HNA containing oligonucleotides synthesis and characterization.

HNA phophoramidites were synthesized according to the literature (1). HNA containing oligonucleotide assembly was performed on an Expedite® 8909 DNA synthesizer (applied Biosystems) by using the phosphoramidite approach. The standard DNA assembly protocols were adjusted for HNA to 600 seconds coupling time. The HNA oligomers were deprotected and cleaved from the solid support by treatment with AMA reagent (NH4OH 33% / methylamine 40%) for two hours at 40°C. After gel filtration on NAP-25® column (Sephadex G25-DNA grade; Pharmacia) with ethanol 10%, the crude mixture was purified using an HPLC system consisted of a Primaide-Hitachi 1110 pump, a Mono Q™ 10/100 column (Pharmacia) ion exchange column, a Uvicord SII 1410 UV detector (Pharmacia-LKB) and a recorder. The oligo sequence was eluted by gradually increasing concentrations of $NaClO_4$ from 10mM to 600mM using buffer B (600mM $NaClO_4$/15% Acetonitrile pH=7). The product-containing fraction was desalted on a NAP-25® column and lyophilized, ready for mass analysis to test purity. Electrospray ionization mass spectra were obtained in negative ion mode on a quadrupole/time-of-flight mass spectrometer (Waters Synapt G2 HDMS, Milford, MA, USA) equipped with a standard ionization source. The instrument resolution was 1.5 × 104 (FWHM) and leucine enkephalin was used as lock mass calibrant. Masses for the oligonucleotides were obtained by deconvolution of the spectra using the MaxEnt algorithm of the software (Waters MassLynx 4.1, Milford, MA, USA). HNA oligonucleotides are listed in Tab. S1 & S2.

SUPPLEMENTARY FIGURES

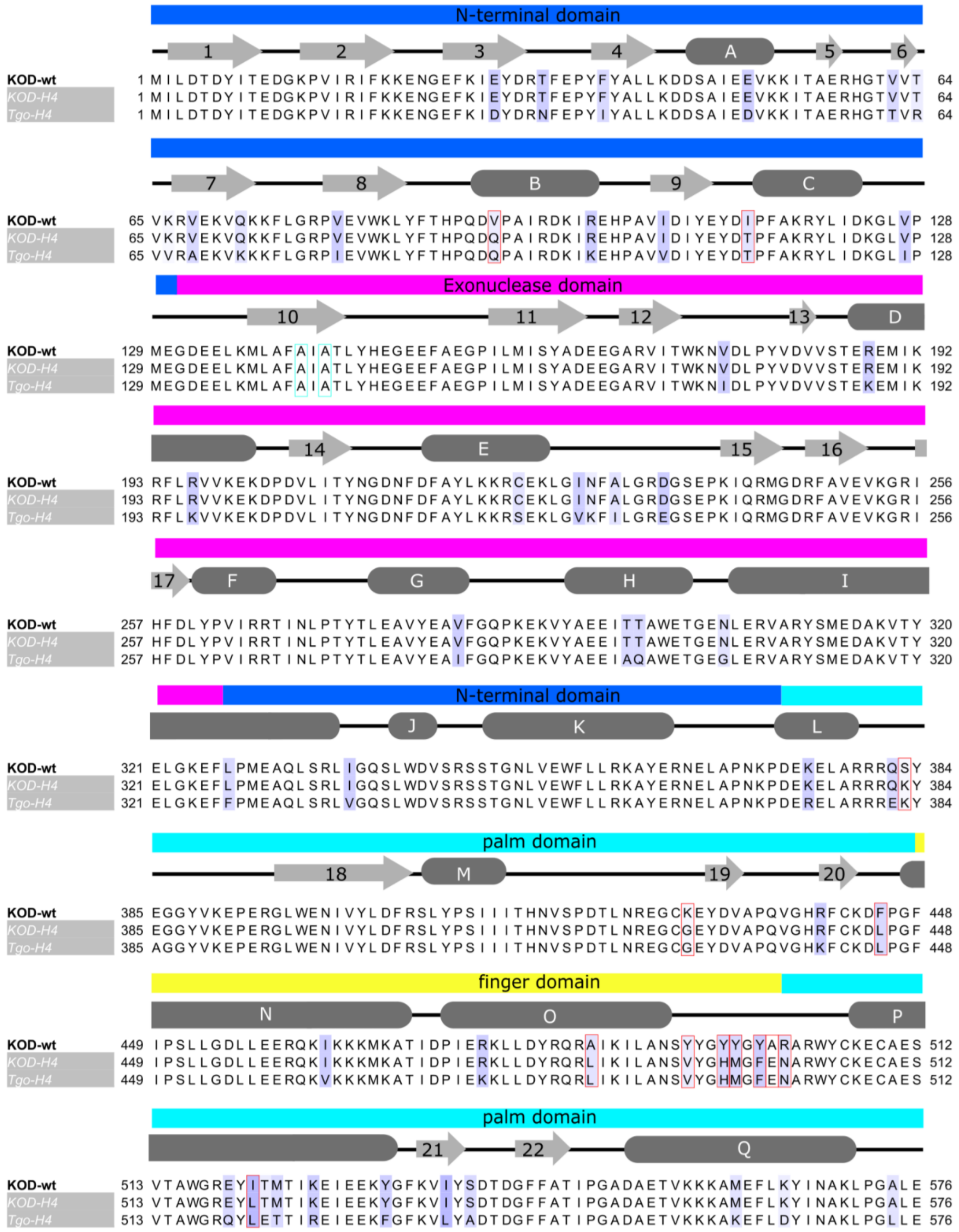

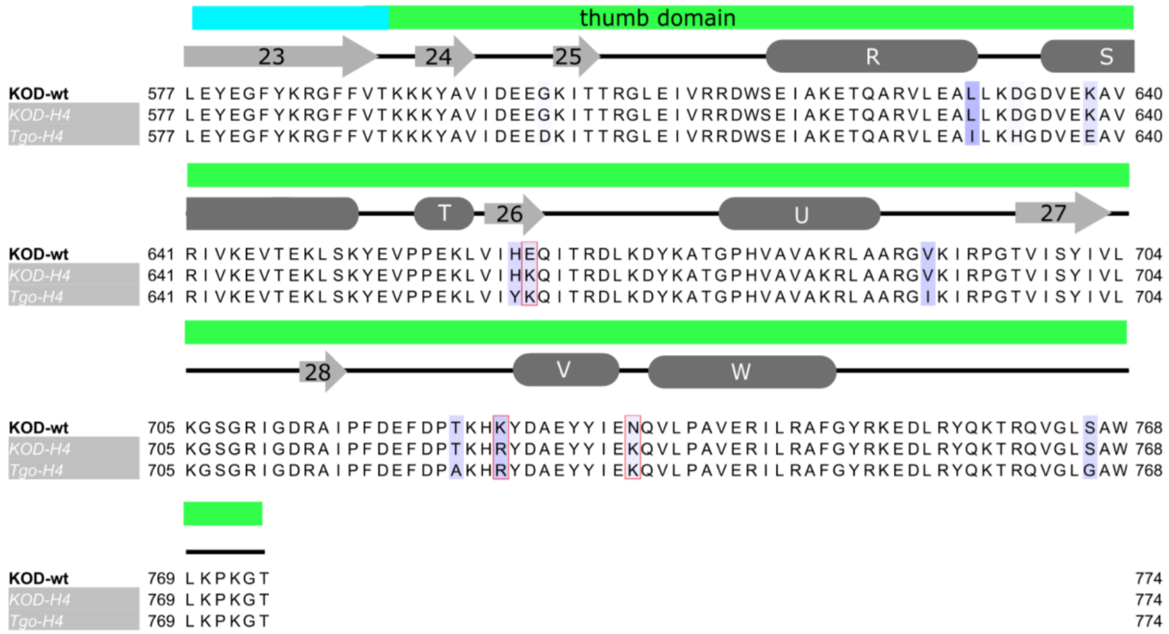

Figure S1. Multiple sequence alignment of KOD-wt, KOD-H4 and Tgo-H4 were adapted from Jalview 2. Subdomains are indicated in different colors: N-terminal (1-130, 327-368), 3′-5′-exonuclease (131-326), palm (369-449, 500-587), finger (450-499) and thumb (588-774)(2). Differences between KOD and Tgo are marked in blue, the exonuclease mutations are framed in cyan, Tgo-H4 mutations are framed in red. Secondary structure elements are named according to KOD-wt (PDB: 48KZ); (3) β-strands are shown as numbered arrows and helices as alphabetical cylinders.

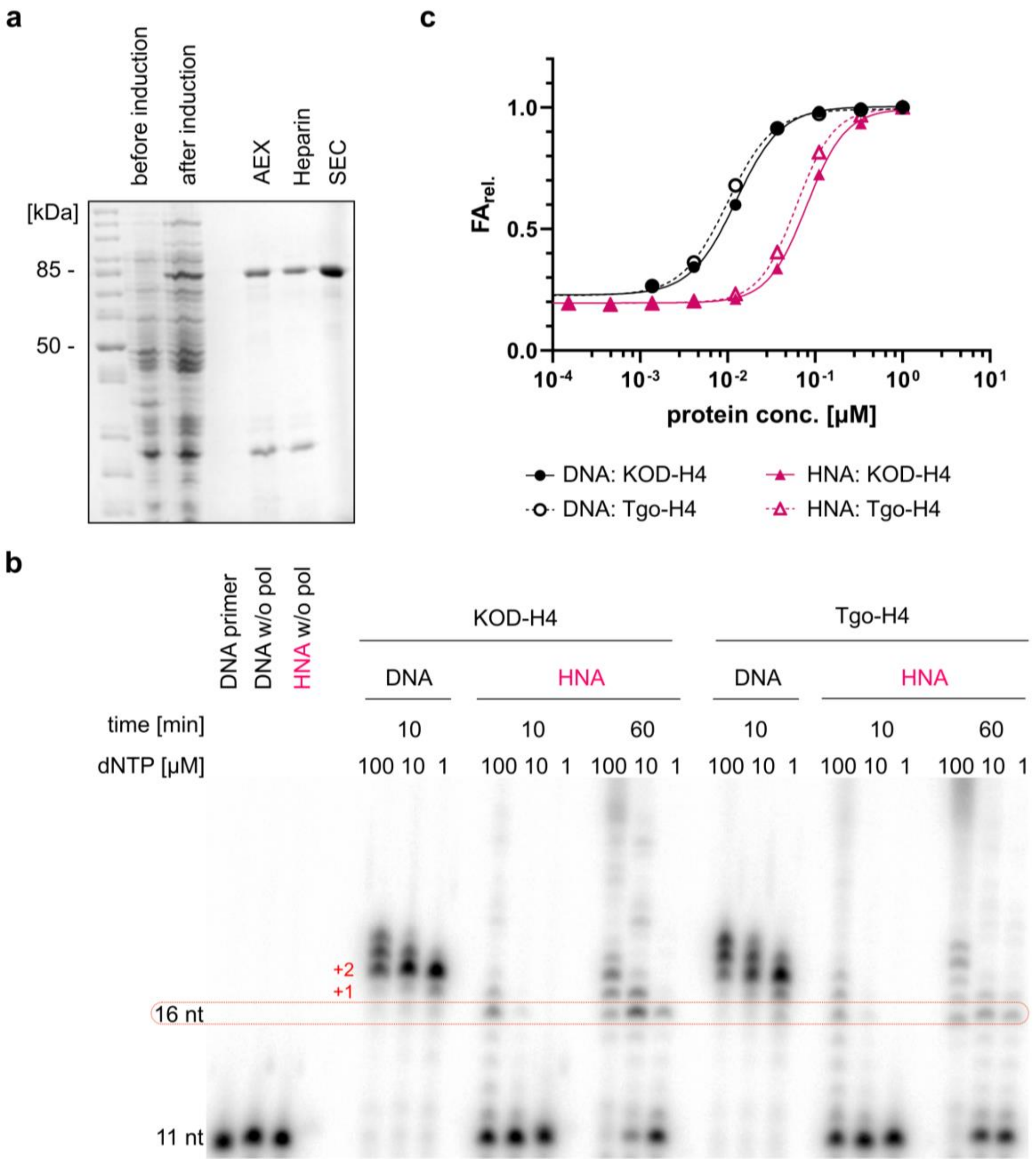

Figure S2. (a) 10% SDS PAGE analysis of KOD-H4 purification stained with Coomassie solution. AEX= anion exchange chromatography, Heparin = elution from Heparin column, SEC = size exclusion chromatography. (b) Radioactive denaturing PAGE analysis of primer extension experiment using KOD-H4 and Tgo-H4 with HNA and DNA templates. Full length product size (16mer oligonucleotide product size = 16 nt, 11 nt = 11mer oligonucleotide primer length) is indicated by a red box. (c) Dose response curve of fluorescence polarization assays of KOD-H4 and Tgo-H4 fitted to normalized log scale for the different templates (DNA, HNA). Fluorescence anisotropy ($FA_{rel.}$) is plotted against the protein concentration [µM]. Analysis was performed using GraphPad Prism version 9.4.1 for Windows, GraphPad Software, San Diego, California USA, www.graphpad.com.

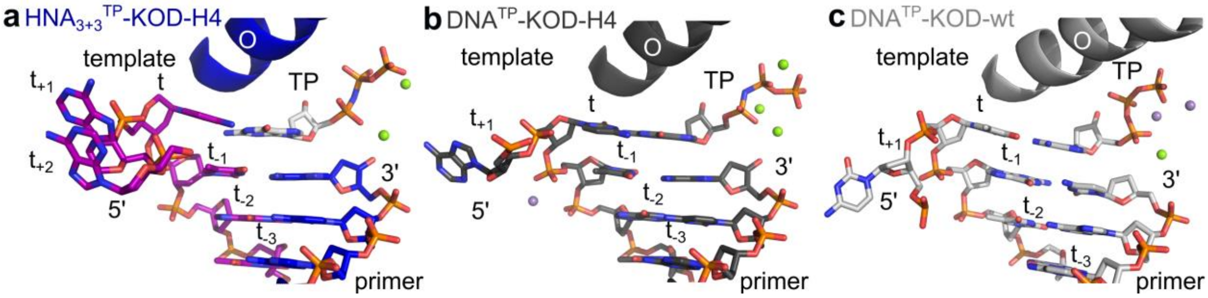

Figure S3. Overview of nucleotides and ions near the active site of KOD-H4 and DNA^{TP}-KOD-wt (5OMF) (4). Nucleotides are shown as sticks in relation to the finger domain O-helix, partially shown as cartoon. HNA nucleotides are color-coded in magenta. Template nucleotides are numbered according to the templating nucleotide (t). Relevant magnesium (green) and manganese (purple) ions are shown as spheres (a) Ternary structure containing six HNA nucleotides: $HNA_{3+3}{}^{TP}$-KOD-H4 (9EMI). (b) DNA ternary structure: DNA^{TP}-KOD-H4 (8S84). (c) Wildtype ternary structure (DNA^{TP}-KOD-wt, 5OMF) (4).

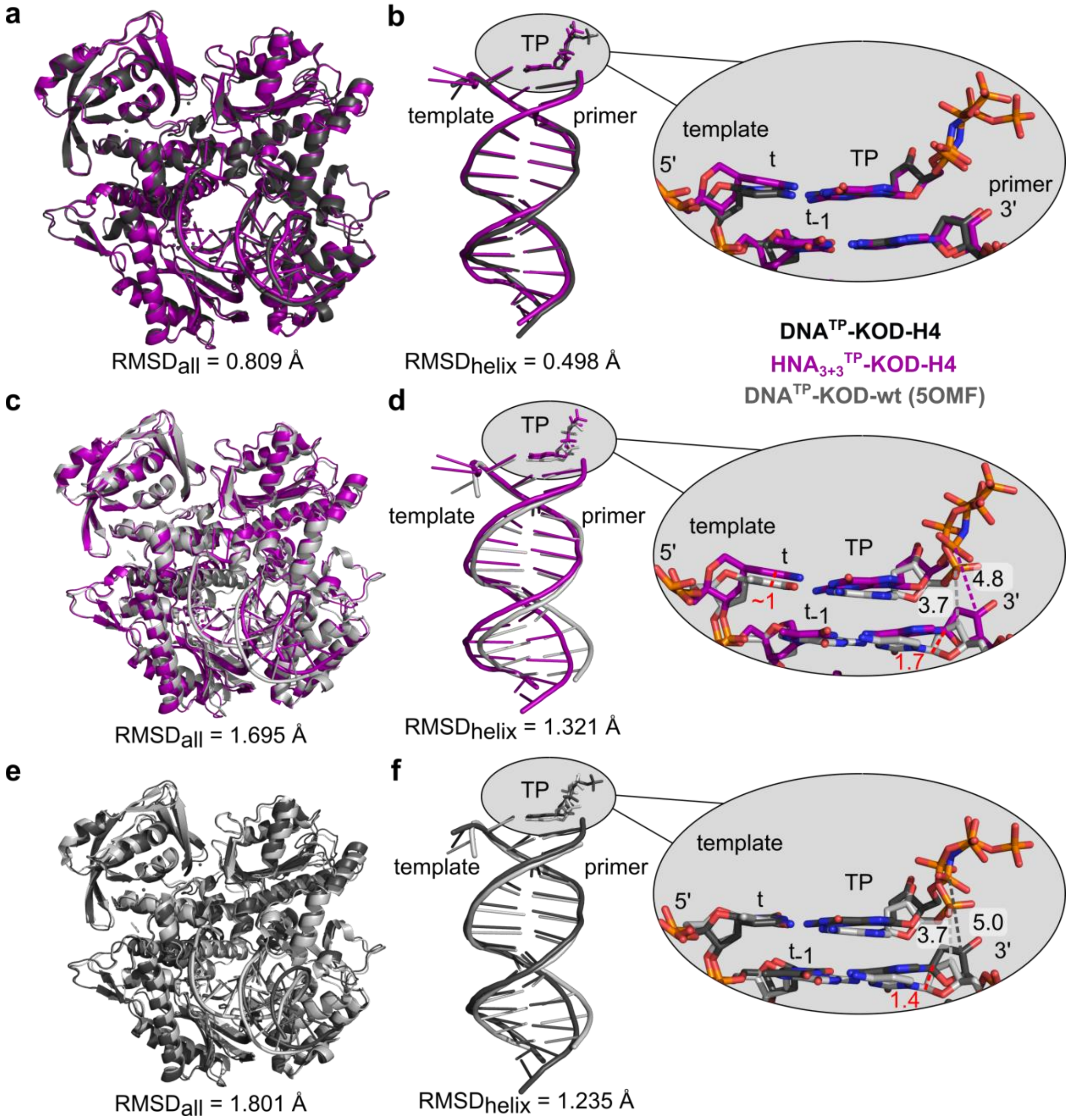

Figure S4. (a, c, e) Pairwise superimposition of DNA^{TP}-KOD-H4 (8S84, dark grey), $HNA_{3+3}{}^{TP}$-KOD-H4 (9EMI, purple) and DNA^{TP}-KOD-wt (5OMF, light grey). Full complexes were superimposed in Pymol and RMSD values are given. (b, d, f) Pairwise superimposition of nucleotide complexes (p/t and TP). In circles, close-ups for the TP binding to the templating nucleotide and the -1 base pair is given. Distances of the primer end to the α-phosphate are indicated and given in Å. Displacements of the terminal primer nucleotides and the templating nucleotides between structures are indicated by red dashed lines and are given in Å.

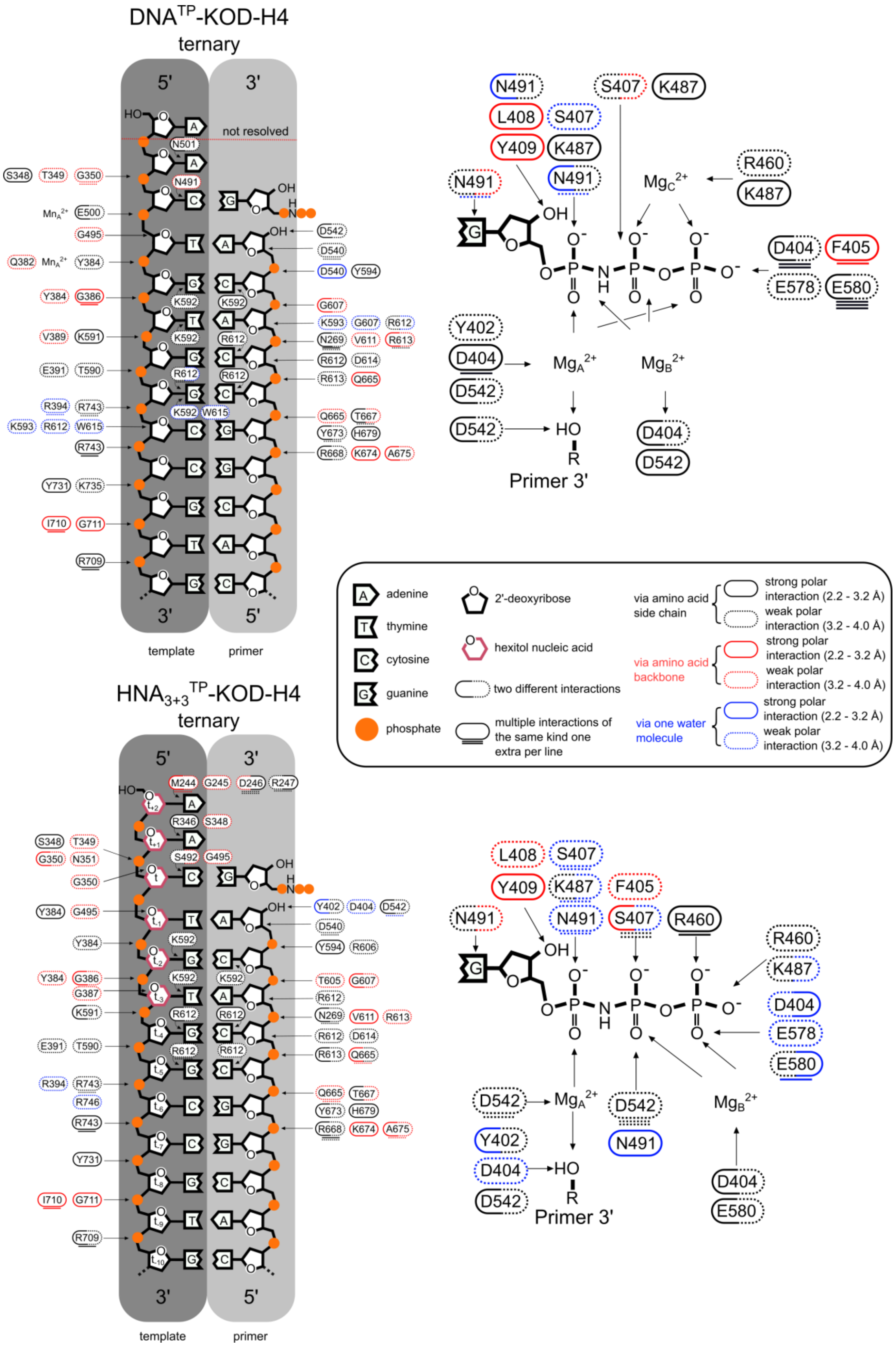

Figure S5. Polar interaction maps of KOD-H4 crystal structures with DNA (top: DNA^{TP}-KOD-H4, 8S84) and HNA (bottom: HNA$_{3+3}$^{TP}-KOD-H4, 9EMI). The underlying criteria and coloring are given in the legend in the figure.

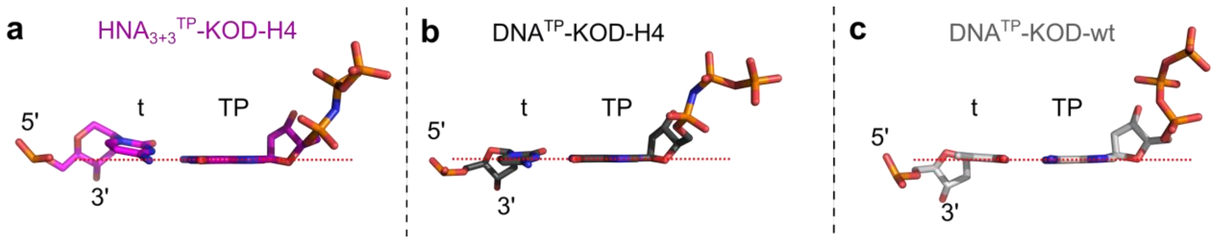

Figure S6. Co-planarity comparison between ternary KOD crystal structures for: (a) HNA_{3+3}^{TP}-KOD-H4 (purple, 9EMI), (b) DNA^{TP}-KOD-H4 (dark grey, 8S84), and (c) DNA^{TP}-KOD-wt (5OMF) (light grey) for the TP binding to the templating nucleotide (t) Plane of the incoming TPs nucleobase is depicted by a dashed red line.

SUPPLEMENTARY TABLES

Table S1. Apparent KD values and Hillslopes given with standard error of the mean (SEM) for KOD-H4 and Tgo-H4 as calculated from fluorescence polarization assays using GraphPad Prism version 9.4.1 for Windows, GraphPad Software, San Diego, California USA, www.graphpad.com.

	KOD-H4		Tgo-H4	
	K_D^{app} ± SEM [nM]	Hillslope ± SEM	K_D^{app} ± SEM [nM]	Hillslope ± SEM
DNA	12.50 ± 0,65	1.67 ± 0,13	10.01 ± 0,31	1.66 ± 0,07
HNA	80.19 ± 6,97	1.95 ± 0,08	61.47 ± 3,07	2.02 ± 0,05

Table S2. Crystallization conditions.

PDB ID	Primer/Template	Crystallization Conditions
8S87	-/- (apo)	15% (w/v) PEG 3K, 20% (v/v) 1,2,4-butantriol, 1% (w/v) NDSB 256, 0.03 M lithium sulfate, 0.03 M sodium sulfate, 0.03 M potassium sulfate, 0.1 M GlyGly/AMPD pH 8.5
9EMI	DNA-Primer/ HNA_{3+3}-Template (ternary)	10% w/v PEG 8000, 20% v/v ethylene glycol 0.02 M D-glucose, 0.02 M D-mannose, 0.02 M D-galactose, 0.02 M L-fucose, 0.02 M D-xylose, 0.02 M N-acetyl-D-glucosamine , 0.1 M bicine/Trizma base pH 8.5
8S84	DNA-Primer/ DNA-Template (ternary)	36 % v/v of a 72% mix: 30% w/v PEG 3000, 40% v/v 1, 2, 4-Butanetriol, 2% w/v NDSB 256; 0.03 M Lithium sulfate, 0.03 M Sodium sulfate, 0.03 Potassium sulfate, 0.1 M MOPSO/Bis-Tris pH 6.5

Table S3. Data collection and refinement statistics. Statistics for the highest-resolution shell are shown in parentheses.

	apo-KOD-H4	HNA_{3+3}^{TP}-KOD-H4	DNA^{TP}-KOD-H4
PDB ID	**8S87**	**9EMI**	**8S84**
Data collection			
Space group	P 2_1 2_1 2_1	P 2_1 2_1 2	P 2_1 2_1 2
a, b, c (Å)	74.42, 110.63, 111.36	111.45, 151.54, 70.40	110.97, 152.46, 70.12
α, β, γ (°)	90, 90, 90	90, 90, 90	90, 90, 90
Resolution range (Å)	44.4 - 1.82 (1.84 - 1.82)	41.04 - 2.27 (2.3 - 2.27)	38.58 - 2.35 (2.38 - 2.35)
R-meas	20.5% (861.9%)	23.3% (418.9%)	26.7% (455.5%)
CC1/2	99.2 (10.8)	99.2 (26.5)	98.6 (10.7)
Mean I/sigma(I)	3.84 (0.17)	7.44 (0.49)	3.52 (0.15)
Completeness (%)	99.40 (96.46)	93.20 (92.19)	97.34 (62.29)
Redundancy	4.71 (4.77)	6.92 (6,20)	3.01 (3.06)
Refinement			
Resolution (Å)	44.58-1.80	46.81-2.27	46.80-2.35
Reflections used in refinement	82546 (2922)	51927 (1852)	49156 (1190)
R_{work}/R_{free}	0.23/0.27	0.23/0.26	0.23/0.27
Number of non hydrogen atoms			
Protein	6217	6214	6782
Nucleic acid duplex /dNTP	-/-	594/31	570/31
Solvent	153	32	14
RMS (bonds) (Å)	0.011	0.003	0.003
RMS (angles) (°)	0.94	0.65	0.64
B-factor			
Average	60.38	73.18	84.48
Protein	60.34	72.58	84.85
Nucleic acid duplex /dNTP	-/-	80.48/59.85	81.61/68.99
Solvent	52.85	64.01	65.84

Table S4. Intraphosphate distances of template strands at positions t to t_{-4} if clear electron density was present. HNA nucleotides are marked in magenta. Distances were measured in Pymol (5).

position	HNA_{3+3}^{TP}-KOD-H4 (9EMI)	DNA^{TP}-KOD-H4 (8S84)	KOD-wt (5OMF)(4)
t-t_{-1}	5.8 Å	6.3 Å	6.0 Å
t_{-1}-t_{-2}	6.0 Å	5.9 Å	6.2 Å
t_{-2}-t_{-3}	6.8 Å	7.0 Å	7.1 Å
t_{-3}-t_{-4}	6.5 Å	6.6 Å	6.5 Å

SUPPLEMENTARY REFERENCES

1. Hendrix, C., Rosemeyer, H., Verheggen, I., Van Aerschot, A., Seela, F. and Herdewijn, P. (1997) 1′, 5′ -Anhydrohexitol Oligonucleotides: Synthesis, Base Pairing and Recognition by Regular Oligodeoxyribonucleotides and Oligoribonucleotides. *Chemistry – A European Journal*, **3**, 110-120.
2. Waterhouse, A.M., Procter, J.B., Martin, D.M.A., Clamp, M. and Barton, G.J. (2009) Jalview Version 2—a multiple sequence alignment editor and analysis workbench. *Bioinformatics*, **25**, 1189-1191.
3. Bergen, K., Betz, K., Welte, W., Diederichs, K. and Marx, A. (2013) Structures of KOD and 9°N DNA Polymerases Complexed with Primer Template Duplex. *ChemBioChem*, **14**, 1058-1062.
4. Kropp, H.M., Betz, K., Wirth, J., Diederichs, K. and Marx, A. (2017) Crystal structures of ternary complexes of archaeal B-family DNA polymerases. *PLOS ONE*, **12**, e0188005.
5. PyMOL: Schrödinger, L., The PyMOL Molecular Graphics System, Version 2.0
